# Coexisting FGFR3 p.K650T mutation in two *FGFR3-TACC3* fusion glioma cases

**DOI:** 10.1186/s40478-019-0721-7

**Published:** 2019-04-29

**Authors:** Leomar Y. Ballester, Soheil Zorofchian Moghadamtousi, Norman E. Leeds, Jason T. Huse, Gregory N. Fuller

**Affiliations:** 1 Molecular Genetic Pathology and Neuropathology, Department of Pathology and Laboratory Medicine, University of Texas Health Science Center, 6431 Fannin St., MSB 2.136, Houston, TX 77030 USA; 2 Vivian L. Smith Department of Neurosurgery, University of Texas Health Science Center, 6431 Fannin St., MSB 2.136, Houston, TX 77030 USA; 3 Memorial Hermann Hospital-TMC, Houston, TX USA; 4 Department of Radiology, University of Texas MD Anderson Cancer Center, Houston, TX 77030 USA

**Keywords:** FGFR3, TERT, Glioblastoma, Glioma, FGFR3-TACC3 fusion, NF1

To the editor,

Chromosomal translocations leading to oncogenic fusions are increasingly being recognized as molecular drivers of gliomas [9]. Approximately 3% of glioblastomas (GBMs) carry a fusion involving fibroblast growth factor receptors (*FGFR1/FGFR3*) with the transforming acidic coiled-coil domains of *TACC1* or *TACC3*. *FGFR3-TACC3* fusion gliomas exhibit characteristic, histologic features, including monomorphous ovoid nuclei, nuclear palisading, perivascular pseudorosettes, an endocrinoid (“chicken-wire”) capillary network, microcalcifications and FGFR3 expression [1]. *FGFR3-TACC3* fusion gliomas are *IDH1/IDH2*-wildtype, and frequently (~ 75% of cases) have *TERT* promoter mutations or *CDKN2A* loss [1]. The histologic features of *FGFR3-TACC3* fusion glioma are similar to those in the recently described polymorphous low-grade neuroepithelial tumor of the young (PLNTY) [2, 4]. In fact, the majority of PLNTYs in some series carry the *FGFR3-TACC3* fusion. Although rare reports of PLNTY occurring in adults exist, the majority of PLNTYs occur in young patients [7]. Although *FGFR3-TACC3* fusion gliomas share histologic features with PLNTY, and may also express CD34, the former typically exhibits high-grade features consonant with glioblastoma and has a mean age at diagnosis of 62 years [1].

Here we describe two cases of infiltrating glioma with histologic features of *FGFR3-TACC3* fusion glioma. Molecular characterization confirmed the presence of *FGFR3*p.K650 T, *TERT* promoter mutation, and *FGFR3-TACC3* fusion in both cases. The cases call attention to an association between the *FGFR3*p.K650 T mutation and *FGFR3-TACC3* fusion.

## Case 1

**Fig. 1:**
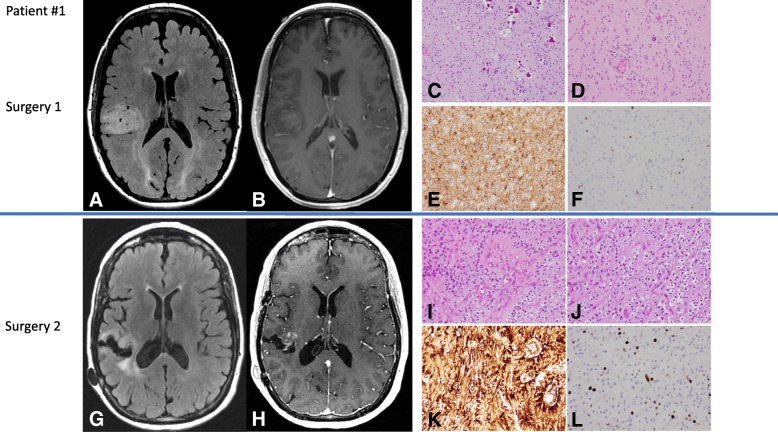
Imaging and histologic features of Patient #1**.** Preoperative MR imaging showing T2 FLAIR hyperintensity in the right temporal lobe (**a**) with minimal contrast enhancement in the T1 sequence with contrast (**b**). H&E-stained tumor tissue sections showing hypercellular brain parenchyma with microcalcifications. The brain parenchyma is infiltrated by monomorphic round glial cells with perinuclear clearing, resembling oligodendrocytes (**c**). Spindle cells in a perivascular distribution are noted (**d**). GFAP is positive in tumor cells (**e**), and Ki67 immunostaining shows a low proliferation index (**f**). Surveillance MR imaging showing T2 FLAIR hyperintensity around the surgical cavity (**g**) and new contrast enhancement along the resection cavity wall (**h**). H&E-stained tumor tissue sections show hypercellular brain parenchyma with oligo-like cells and perivascular pseudorosettes (**i**), and an endocrinoid (“chickenwire”) capillary network (**j**). The tumor cells are strongly positive for CD34 (**k**) and the Ki67 index is elevated (**l**) in comparison to the tumor resected in the first surgery (**d**)

A 51-year-old woman presented with a 2-year history of numbness and left arm pain, with negative spine imaging and peripheral neuropathy workup. Due to new paresthesia of the left hip, MR imaging (MRI) of the brain was performed, which showed T2 hyperintensity in the right insula, associated with edema and mild contrast enhancement. Microscopic examination of the resected tumor showed hypercellular brain parenchyma infiltrated by small round monomorphic cells with perinuclear clearing resembling oligodendroglioma, microcalcifications and perivascular pseudorosettes. Mitotic activity was inconspicuous, and computer-assisted quantitation yielded a Ki67 proliferation index of 7.6% (Fig. 1). A diagnosis of oligodendroglioma, NOS, WHO grade II, was rendered following guidelines from the 2007 WHO classification system for tumors of the central nervous system (CNS), which was in force at the time of diagnosis. Fluorescence in situ hybridization (FISH) analysis for chromosomal arms 1p and 19q was negative for codeletion. The patient was treated with intensity-modulated radiation therapy (IMRT) to a total dose of 50.4Gy in 28 fractions, together with 12 cycles of temozolomide chemotherapy.

**Table 1:** Genetic alterations identified by NGS

	Surgery 1 Infiltrating glioma, WHO grade II	Surgery 2 Glioblastoma, WHO grade IV
Patient 1	FGFR3 p.K650 T (MAF = 4%) FGFR3-TACC3 fusion	FGFR3 p.K650 T (MAF = 29%) NF1 p.F443C TERT c.-124C > T FGFR3-TACC3 (COSF1353)
	N/A	Surgery 1 Glioblastoma, WHO grade IV
Patient 2	N/A	FGFR3 p.K650 T (MAF = 12%) TERT c.-146C > T FGFR3-TACC3 (COSF1348)

The patient was placed on surveillance imaging every 3 months and was stable until ~ 3 years after presentation when a new area of contrast enhancement was identified adjacent to the resection cavity. Resection of the recurrent lesion was performed. Microscopic examination showed a compact, densely cellular glioma with morphologic features associated with the recently-described *FGFR3-TACC3* fusion glioma [3, 6, 9]. The characteristic features evident in this case include a population of glioma cells with monomorphous ovoid nuclei, nuclear palisading and enfilading, thin parallel cytoplasmic processes, endocrinoid capillary network, microcalcifications and desmoplasia (Fig. 1) [1]. The tumor from the second resection showed foci of vascular proliferation, correlating with the presence of contrast enhancement on the preoperative MRI. In contrast to the low proliferation index of the initial tumor, the recurrent tumor showed a Ki67 index of 30.3%. GFAP was expressed in perivascular cell processes of the tumor cells, EMA was negative, and expression of the ATRX protein was retained.

Next generation sequencing analysis (NGS) for mutations (134 genes), copy number variations (47 genes), and fusions (51 genes), was performed on the recurrent tumor. The results showed *FGFR3*p.K650 T, *NF1*p.F443C and *TERT*c.-124C > T mutations, as well as the *FGFR3-TACC3* (COSF1353) fusion. These findings prompted analysis of the initial tumor. NGS analysis revealed only the presence of *FGFR3*p.K650 T mutation; *NF1* and *TERT* mutations were not identified in the tumor from the first surgery (Table 1). Conventional RT-PCR with *FGFR3* and *TACC3* specific primers (5′-AGGAGCTCTTCAAGCTGCTG-3′ and 5′-GGGGGTCGAACTTGAGGTAT-3′) generated a product of the expected size (225 bp) and confirmed the *FGFR3-TACC3* fusion in the original tumor.

## Case 2

**Fig. 2:**
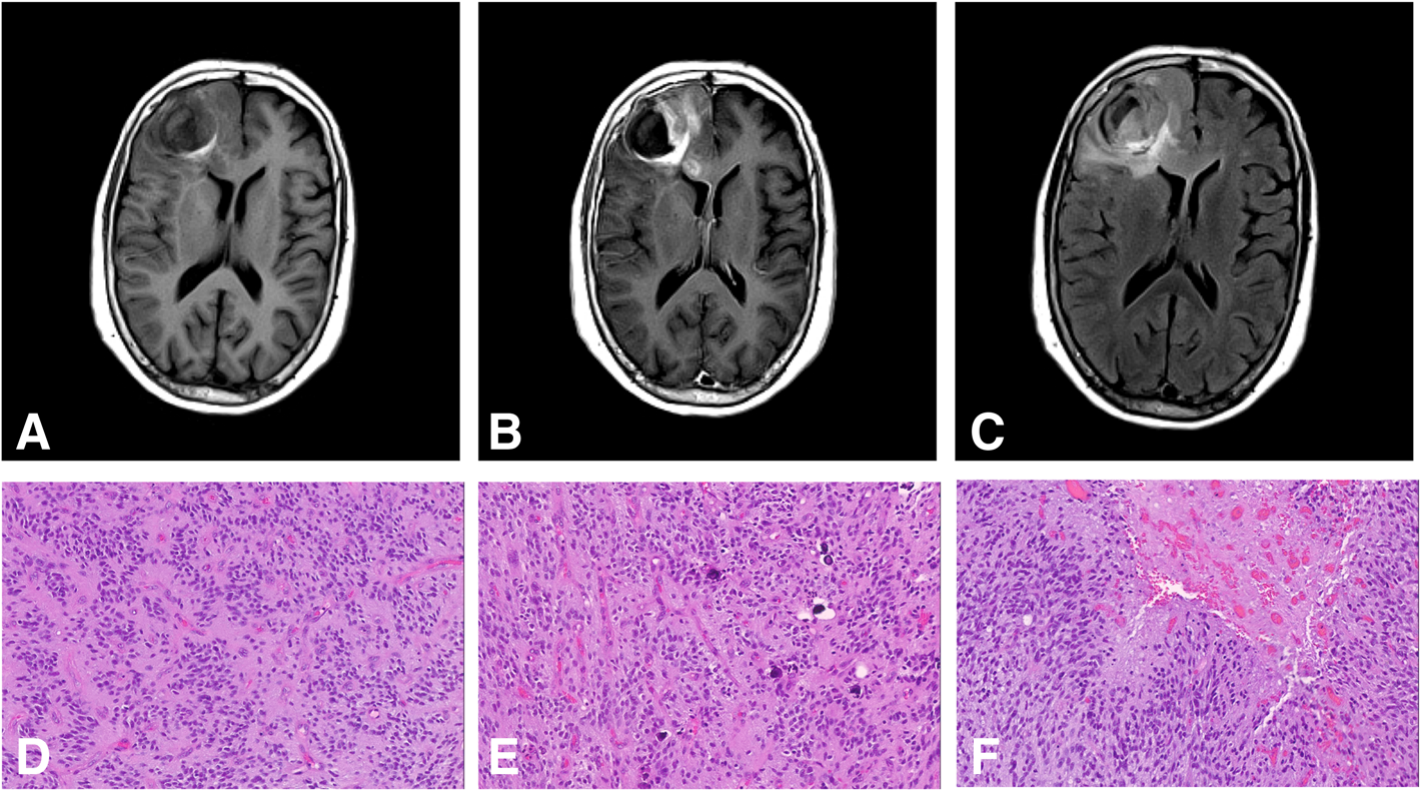
Imaging and histologic features of Patient #2. **a** Preoperative MR imaging (axial T1 without contrast) showing a lesion in the right frontal lobe with an area of intrinsic T1 hyperintensity. **b** T1 with contrast image showing areas of contrast enhancement associated with the right frontal lobe lesion. **c** T2-FLAIR sequence showing a large area of T2 hyperintensity in the right frontal lobe, partially extending into the corpus callosum. **d** Microscopic sections of the tumor showing hypercellular brain parenchyma with glial tumor cells forming perivascular pseudorosettes. **e** Areas of the tumor showed an endocrinoid capillary network and microcalcifications. **f** Necrosis with pseudopalisading, consistent with glioblastoma, is also present

A 75-year-old female presented to our institution for evaluation of treatment options after a diagnosis of malignant glioma. H&E-stained sections from the biopsy showed a high-grade glial tumor with microcalcifications, perivascular pseudorosettes, elevated mitotic activity, vascular proliferation and necrosis with pseudopalisading (Fig. 2). The tumor cells expressed GFAP, punctate EMA staining was present in several areas, and automated quantitation yielded a Ki67 proliferation index of ~ 50%. The final diagnosis was glioblastoma, IDH-wildtype, WHO grade IV, based on the 2016 WHO classification of CNS tumors. Subsequent NGS analysis (same assay as described above) showed the presence of *FGFR3*p.K650 T and *TERT*c.-146C > T mutations, and an *FGFR3-TACC3* fusion (COSF1348). The patient was treated with concurrent radiation and temozolomide.

These cases illustrate the morphologic and molecular alterations of *FGFR3-TACC3* fusion glioma. The molecular alterations emphasize several important points. (1) The strong association between *FGFR3-TACC3* fusion and *TERT* promoter mutations. (2) The novel concurrent association between the *FGFR3*p.K650 T point mutation and the *FGFR3-TACC3* fusion. This association has clinical importance because analysis for gene mutations is more commonly done than fusion analysis. The presence of *FGFR3*p.K650 T should alert the physician to the possibility of an *FGFR3-TACC3* fusion. Previous studies of *FGFR3-TACC3* fusion gliomas have focused on fusion detection, with minimal simultaneous mutation analysis. It is therefore unclear if *FGFR3*p.K650 T is uniformly associated with the *FGFR3-TACC3* fusion, although these examples suggest that may be the case. Given the existence of FGFR inhibitors, some which are being evaluated for the treatment of glioblastoma, recognition of the association between these two molecular alterations is important [5]. (3) The case data presented here supports a potential pathway to progression in *FGFR3-TACC3* fusion glioma, from low-grade to high-grade, through acquisition of a *TERT* promoter mutation.

*FGFR3* alterations are common in bladder cancers (~ 35% of cases) but extremely rare in brain tumors. *FGFR3* codon 650 is 1 of 3 somatic mutation hotspots in this gene, although it is the least commonly affected (COSMIC). In COSMIC, there are ~ 200 reports of mutations at FGFR3p.650; in total there are 3 high-grade astrocytomas with an FGFR3 p.K650 T mutation. The FGFR3 p.K650 T mutation is reported as a pathogenic mutation in Clinvar and GeneReviews. Also, the FATHMM in silico method gives this mutation a pathogenic score of 0.98; scores of > 0.7 are considered pathogenic and are reported as such in COSMIC. We were unable to find reports with functional data on the FGFR3 p.K650 T mutation. However, FGFR3 p.K650**E** has been associated with constitutive activation of the receptor [8].

There is a noteworthy similarity between the histology of PLNTY and *FGFR3-TACC3* fusion glioma. There are also similarities in their genetic alterations, given that the *FGFR3-TACC3* fusion has been described in both entities. However, there is a significant difference in the mean age at diagnosis between PLNTY and *FGFR3-TACC3* fusion glioma, being 17.6 years (4–32) vs. 67 (35–87) years, respectively. Also, *TERT* promoter mutations and *CDNK2A* loss frequently occur in *FGFR3-TACC3* fusion gliomas, whereas these alterations have not been reported in PLNTY.

Suspicion for the *FGFR3-TACC3* fusion in the two present cases began with microscopic examination of H&E-stained sections, which elicited fusion testing. Recognition of the characteristic histologic feature set of *FGFR3-TACC3* fusion glioma (and PLNTY) prompted molecular testing in both cases. In an era in which stratification and treatment of brain tumors is increasingly being guided by molecular information, recognizing the possibility of an *FGFR3-TACC3* fusion in an infiltrating astrocytoma is critical, and may result in significant therapeutic impact.
